# The Potential Regulatory Role of Ferroptosis in Orthodontically Induced Inflammatory Root Resorption

**DOI:** 10.3390/ijms252413617

**Published:** 2024-12-19

**Authors:** Leilei Wang, Chuan Wang, Hong He

**Affiliations:** 1 State Key Laboratory of Oral & Maxillofacial Reconstruction and Regeneration, Key Laboratory of Oral Biomedicine Ministry of Education, Hubei Key Laboratory of Stomatology, School & Hospital of Stomatology, Wuhan University, Wuhan 430079, China; 2 Department of Periodontology, School & Hospital of Stomatology, Wuhan University, Wuhan 430079, China; 3 Department of Orthodontics, School & Hospital of Stomatology, Wuhan University, Wuhan 430079, China

**Keywords:** ferroptosis, orthodontic tooth movement, orthodontically induced inflammatory root resorption, inflammation

## Abstract

People, in increasing numbers, are seeking orthodontic treatment to correct malocclusion, while some of them are suffering from orthodontically induced inflammatory root resorption (OIIRR). Recent evidence suggests that the immune-inflammatory response occurring during bone remodeling may be responsible for OIIRR. Ferroptosis, a new type of programmed cell death (PCD), has been found to have a close interrelation with inflammation during disease progression. While ferroptosis has been extensively studied in bone-related diseases, its role in OIIRR is poorly understood. Considering that the tooth root shares a lot of similar characteristics with bone, it is reasonable to hypothesize that ferroptosis contributes to the development of OIIRR. Nevertheless, direct evidence supporting this theory is currently lacking. In this review, we introduced ferroptosis and elucidated the mechanisms underlying orthodontic tooth movement (OTM) and OIIRR, with a special focus on the pivotal role inflammation plays in these processes. Additionally, we covered recent research exploring the connections between inflammation and ferroptosis. Lastly, we emphasized the important regulatory function of ferroptosis in bone homeostasis. Further investigations are required to clarify the modulation mechanisms of ferroptosis in OIIRR and to develop novel and potential therapeutic strategies for the management of OIIRR.

## 1. Introduction

Orthodontic tooth movement (OTM) is initiated by a mechanical force that triggers bone resorption and apposition [1]. Approximately 80% of orthodontic patients may experience orthodontically induced inflammatory root resorption (OIIRR) [2], with some severe cases potentially leading to tooth mobility and even tooth loss [3]. Among the various external and individual factors, the inflammatory mechanism has recently been proven to be a potential factor for OIIRR [4].

Ever since ferroptosis was first coined as a type of programmed cell death (PCD) in 2012, this iron-dependent cell death process has been regulated by reactive oxygen species (ROS) accumulation and lipid peroxidation. It has been extensively researched for the past decade in order to understand its relationship with various diseases, including metabolic diseases [5] and cancers [6,7,8]. Compelling evidence demonstrates that ferroptosis plays an important role in inflammatory bone diseases such as osteoporosis [9] and osteoarthritis [10]. Despite the many similarities between the tooth root and bone, the molecular mechanisms of ferroptosis in OIIRR remain unclear.

In this review, we comprehensively summarize ferroptosis, and we also explore the mechanism of OTM and OIIRR, emphasizing the role of inflammation in this process. Moreover, we discuss recent studies examining the relationships between inflammation and ferroptosis, as well as between autophagy and ferroptosis. Additionally, we highlight the regulatory role of ferroptosis in bone homeostasis. We aim to unfold the potential evidence regarding the cellular and molecular mechanisms through which ferroptosis influences OIIRR. Importantly, we propose the possible application of ferroptosis regulators to modulate OIIRR, hoping to provide a novel insight for preventing unwanted side effects during OTM.

## 2. Introduction to Ferroptosis

Ferroptosis is a type of PCD that distinguishes itself from other forms of cell death like apoptosis and necrosis [11]. Apoptosis is characterized by nuclear pyknosis, cell shrinkage, and DNA fragmentation, whereas necrosis exhibits features including cell swelling, organelle disintegration and protein denaturation. In contrast, the process of ferroptosis is defined by the iron-dependent accumulation of lethal lipid species derived from lipid peroxidation, a process that can be prevented by antioxidant systems, notably glutathione peroxidase 4 (GPX4) and ferroptosis suppressor protein 1 (FSP1) [12].

Iron accumulation plays a vital role in the process of ferroptosis. Maintaining homeostasis requires the normal operation of iron metabolism including iron uptake, storage, utilization, and excretion. Fe^3+^ in the serum binds to transferrin (TF), which is then recognized and endocytosed by the transferrin receptor (TFRC/TfR1). Subsequently, the Fe^3+^ is immediately reduced to Fe^2+^ by the metal reductase six-transmembrane epithelial antigen of prostate 3 (STEAP3), which enters the cytoplasm with the assistance of the bivalent metal transporter divalent metal transporter 1 (DMT1). Mostly, the intracellular iron is stored in the form of ferritin, a common ferri-binding protein. Ferritin can be delivered to the autophagosomes by nuclear receptor coactivator 4 (NCOA4). Ferritin then degrades and releases free iron in the autophagosomes. This process is named ferritinophagy, and it increases the sensitivity of ferroptosis due to the intracellular ferrous iron [13]. The excess Fe^2+^ reacts with H_2_O_2_ to produce hydroxyl radicals through the Fenton reaction. The product is capable of damaging DNA, lipid membranes, and other biomolecules.

Another process that initiates ferroptosis is lipid peroxidation. Free iron catalyzes the generation of excessive levels of ROS [14]. These ROS produce peroxy free radical intermediates that can react with lipids to form lipid peroxides (LOOHs). Normally, cells possess detoxifying agents such as GPX4 that reduce lipid peroxides to fatty alcohols, thereby protecting cells. However, when the ROS is too much to eliminate, or the oxidative imbalance happens under pathological conditions, ferroptosis occurs.

## 3. Orthodontic Tooth Movement and Root Resorption

### 3.1. Orthodontic Tooth Movement: Biological Mechanism

In this part, we describe the classical pressure–tension theory to demonstrate the role of bone cells and chemical mediators, especially inflammatory cytokines, on OTM. In addition, new evidence that explains the mechanisms of OTM have also been elaborated, which includes NcRNAs and immune responses.

#### 3.1.1. Classical Pressure–Tension Theory

The classical pressure–tension theory proposes that tooth movement is initiated by chemical stimuli. Within just a few seconds of force application, the tooth shifts its position within the periodontal ligament (PDL) space, resulting in PDL compression or stretch. With sustained application of force, the oxygen tension and the chemical mediators including prostaglandins and cytokines (e.g., Interleukin (IL)-1β) in the blood flow undergo changes within minutes. These changes result in bone formation at the tension side and bone resorption at the compression side. On the compression side, heavy force cuts off blood flow and causes cell death (hyalinization), thus no osteoclast differentiation occurs within the compressed PDL space, and this facilitates tooth movement.

#### 3.1.2. Emerging Evidence for the Biological Mechanisms of OTM

Recently, emerging evidence provides alternative explanations for the classical pressure–tension theory. Studies have shown that myofibroblasts, intracellular Ca^2+^-regulated biorhythm, hypoxia, autophagy, and noncoding RNAs (ncRNAs) might be involved in this process [15]. NcRNAs, which do not translate into proteins, consist of long ncRNAs (lncRNAs), microRNAs (miRNAs), transfer RNAs (tRNAs), and ribosomal RNAs (rRNAs). Orthodontic loading triggers shear, tension, and compressive deformation in the heterogeneous periodontium, altering the expression level of ncRNAs in the effector cells. These ncRNAs can modulate numerous signaling pathways like Mitogen-activated protein kinase (MAPK), WNT and Phosphatidylinositol-3-kinase/serine-threonine kinase (PI3K/Akt), thereby triggering autophagy, cell proliferation, differentiation, and immune-inflammatory responses. These mechanisms collectively regulate the mechanobiological process of OTM [16,17]. Taken together, ncRNAs have the potential to serve as biomarkers for exploring orthodontic optimal forces, enhancing clinical outcomes, and preventing root resorption.

It is proposed that orthodontic force elicits systemic immune responses within periodontal tissues, followed by the recruitment of the systemic inflammatory monocytes and multiple inflammatory factors [18]. Immune cells like macrophages, T cells, and B cells have been recently reported to regulate osteogenesis and osteoclastogenesis. Researchers are making efforts to find approaches to inhibit odontoclast activities or enhance cementoblast activities to prevent orthodontic root resorption. Notably, circadian rhythm has been proven to influence bone and periodontal remodeling, as the expression of the osteogenic genes and the protein releases exhibit a circadian trend [19]. This reminds us to focus more on the force loading timing and set up a periodicity pattern of orthodontic traction at night during orthodontic treatment. To sum up, these novel investigation approaches and trends may provide an abundant biological foundation for the experimental and clinical interventions for OTM.

In conclusion, OTM is the fundamental process for orthodontic treatment. It is initiated by the orthodontic force, which is converted into biochemical signals, that activate mechano-sensitive genes and various signaling pathways in various cell types surrounding the teeth. These signals change the functions of the cells and ultimately facilitate the movement of the tooth. NcRNAs, circadian rhythm and other regulators modulate the translation of the signals to ensure the accuracy of the whole process. Notably, the involvement of immune cells indicates that an immune-inflammatory response may play an essential role during OTM.

### 3.2. How Root Resorption Occurs During Orthodontic Tooth Movement

#### 3.2.1. Role of Inflammation in Orthodontic Tooth Movement

As the theory of OTM describes it, tooth loading causes a distortion of PDL nerve endings, leading to the release of vasoactive neurotransmitters to interact with vascular endothelial cells, causing vasodilation and increased permeability with plasma leakage. Then, the activated endothelium recruits circulating leukocytes, monocytes, and macrophages to the PDL, marking the onset of acute inflammation. Several days later, the acute inflammation transitions to a chronic and proliferative process that involve fibroblasts, endothelial cells, osteoblasts, and osteoclasts. During this process, native periodontal cells, leukocytes, and platelets release various inflammatory factors to initiate the remodeling of bone and paradental tissues. These factors include cytokines such as prostaglandins (PGs), IL-1β, tumor necrosis factor-α (TNF-α), nitric oxide (NO), IL-10, and transforming growth factor β (TGF-β), whose roles will be elaborated in the following paragraph.

Compression and tension areas are associated with corresponding mediators regulating resorption and deposition, respectively. Compression leads to increased cycloxygenase-2 (COX-2) that catalyzes the production of PGs, including prostaglandin E2 (PGE2). PGs can increase the concentration of intracellular cyclic adenosine monophosphate (cAMP) in osteoclasts and boost their resorptive activity, while PGE2 can stimulate the differentiation and expression of RANKL/OPG in osteoblasts [20]. Furthermore, the increased RANKL and decreased OPG in osteoblasts favors osteoclast differentiation and increases bone resorption. In addition, IL-1β and TNF-α can induce osteoclast differentiation and survival, further stimulating inflammation and matrix metalloprotease (MMP) levels. The MMPs degrade PDL extracellular matrix and the boney organic matrix, enabling the attachment of osteoclasts for bone resorption [21]. Once osteoclasts remove all of the necrotic tissue, tooth movement begins. Osteoblasts then create osteoid with new periodontal fibrils embedded in the alveolar bone wall and root cementum. Moreover, compression-induced bone morphogenic protein (BMPs) and Runx2 strengthen osteoblast differentiation and bone mineralization, while active fibroblasts upregulate extracellular matrix fiber production. Subsequently, the compressed bone and PDL are disassembled and rebuilt.

In the tension zone, tensile strain stimulates the proliferation of osteoblast progenitor in the PDL and activates endothelial nitric oxide synthase (eNOS) to produce NO, which mediates bone formation. Cytokine IL-10 increases in tension areas, boosting OPG and reducing RANKL production by osteoblasts, thereby inhibiting osteoclast formation, activity, and survival. Moreover, stimulated TGF-β recruits osteoblast precursors, induces their differentiation, downregulates MMPs, and upregulates tissue inhibitors of metalloproteases (TIMPs). MMPs and TIMPs work together to regulate bone remodeling [22]. The cumulative results increase osteoblast and reduce osteoclast activity, leading to bone production and remodeled PDL fibers on the opposite side of tooth movement.

In short, inflammatory cytokines, immune cells, and specific immune signaling pathways play pivotal roles in regulating different bone cells and therefore causes tooth movement. However, unwanted resorption occurs frequently on the tooth root, even though the whole process is precisely controlled under normal conditions (i.e., appropriate force, without infection, etc.). While considerable research has been conducted to elucidate the underlying mechanisms, further investigations are still necessary to fully comprehend this complex process.

#### 3.2.2. Orthodontically Induced Inflammatory Root Resorption (OIIRR) and Inflammation

Alveolar bone undergoes remodeling in response to force, while tooth root is protected by the cementum. Nevertheless, OIIRR still occurs on mineralized cementum or dentine in the presence of osteoclast-like multi-/mono-nucleated cells [23]. OIIRR, as a destruction of root structure due to sterile inflammation, is one of the most frequently reported side effects following orthodontic treatment. Radiographic studies indicate that 48–66% of orthodontically treated teeth experience root resorption of 2 mm or less, and histological findings reveal that around 90% of the orthodontic cases experienced root resorption [24]. Although several protective procedures (e.g., using light-force wires as initial wires) have been suggested, none of them could truly prevent OIIRR with any degree of certainty, and no unequivocal evidence is yet available referring to the underlying mechanism and biological factors that trigger OIIRR.

**Figure 1:**
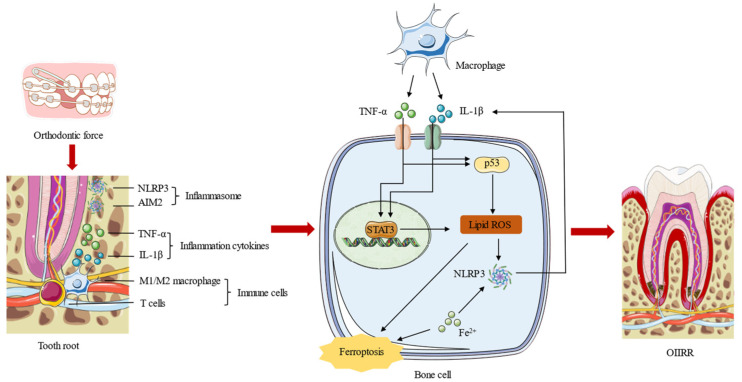
Orthodontic force-induced inflammation may cause root resorption by inducing ferroptosis. The inflammation reactions commence immediately after orthodontic force is applied to the teeth. Inflammatory cytokines, including TNF-α and IL-1β secreted by immune cells, can cause lipid ROS accumulation through stat3 or p53, ultimately inducing ferroptosis. In addition, the NLRP3 inflammasome can trigger the release of IL-1β to induce ferroptosis. Iron and lipid peroxidation can also induce ferroptosis. Ferroptosis of different types of bone cells can result in alveolar bone remodeling, resulting in tooth resorption. Black arrows represent promotion, and red arrows represent the progress of OIIRR.

As described above, inflammation plays a crucial role in OTM, and it could lead to tooth destruction when left uncontrolled. The mechanisms for the inflammation-mediated tooth movement or root resorption may be similar. Bone remodeling is a complex process modulated by multiple factors including inflammation cytokines, immune cells, and the inflammasome, and these immune-inflammatory responses may simultaneously lead to root resorption (Figure 1), which will be discussed in the following context.

Soluble mediators, including cytokines secreted by immune cells within the periodontal tissues, are responsible for root resorption when teeth are exposed to heavy orthodontic force. These inflammatory mediators include interleukins, prostaglandins, TNF-superfamily, and RANK/RANKL/OPG. Notably, the significance of proinflammatory cytokines like TNF-α and IL-1β has been documented. For instance, OTM was reduced in the TNF-α^−/−^ mice [25]. Moreover, the expression level of IL-1β was increased in the gingival crevicular fluid (GCF) of patients undergoing orthodontic treatment, promoting osteoclastogenesis [26]. On the contrary, increased root resorption was observed in IL-1β knock-out mice [27], and people with a lower IL-1β production genotype exhibit a higher risk of root resorption [28]. The reason for this paradoxical effect of IL-1β deficiency remains challenging to explain. One theory is that the lack of IL-1β inhibits alveolar bone remodeling, leading to the long-time stress concentrated on the tooth root, ultimately causing the destruction of cementum [29].

The immune-inflammatory response exerts multiple effects on different stages of OTM [30]. Some immune cells participate in the process of root resorption during orthodontic treatment, while others influence bone remodeling. The increased M1/M2 phenotype ratio of macrophages results in root resorption [31]. In addition, helper T cells are able to aggravate root resorption [32]. Moreover, systemic inflammatory monocytes could be recruited to periodontal tissues by orthodontic force stimulus, which may provide a novel insight into the relationship between monocytes with root resorption [18]. Furthermore, PD-L1 is expressed on OIIRR-associated cell types in periodontal tissue, including osteoblasts, osteoclasts, PDL cells, and gingival fibroblasts. This expression may reveal the possible recognition mechanism of PD-L1 for immune cells during OIIRR [33].

The inflammasome is a protein complex that mainly contains the receptor, the adaptor apoptosis-associated speck-like protein containing CARD (ASC) and downstream caspase-1. Upon the activation of the receptor by an agonist, ASC assembles into an inflammasome with a diameter reaching microns, thereby inducing caspase-1 to self-cleave and activate. This activated caspase-1 promotes the maturation and secretion of several pro-inflammatory cytokines, including IL-1β and IL-18. The overexpression of the NOD-like receptor thermal protein domain associated protein 3 (NLRP3) inflammasome in osteoblasts, osteoclasts, periodontal ligament fibroblasts, and leukocytes is associated with cellular dysfunction and environmental abnormality, leading to the disorganization of ligament and alveolar bone [34]. Inflammatory bone resorption during OTM is triggered by mechanical stress. Thus, inflammatory cytokines such as IL-1β, which are crucial to induce inflammasome activation, could be upregulated by the increased orthodontic force in humans. In rat models loaded with excessive orthodontic force, the expression levels of NLRP3 and caspase-1 are upregulated [35]. Moreover, in diabetes mellitus rats that received orthodontic treatment, NLRP3 was involved in diabetes-induced periodontal changes [36]. In regards to cyclic stretching, the levels of NLRP3 are increased in hPDLCs [37], but are suppressed in macrophages [38]. Furthermore, a cross-sectional study suggests that the absent in melanoma 2 (AIM2) inflammasome is associated with alveolar bone loss in patients with periodontitis [39]. These findings suggest that the inflammasome is associated with bone remodeling in response to orthodontic mechanical force, although its effects on OIIRR need further study.

In summary, OIIRR is a common occurrence during orthodontic treatment, while the underlying mechanism remains unclear. Current evidence suggest that the immune-inflammatory response that occurs during bone remodeling may be responsible for OIIRR. Since inflammation is a highly complicated process that involves multiple signaling pathways and regulatory factors, further investigation is required to identify the relationship between OIIRR and inflammation, aiming for better prevention and treatment strategies for OIIRR.

## 4. The Possible Modulation Mechanisms of Ferroptosis on OIIRR

### 4.1. The Bridge Between Ferroptosis and OIIRR—Inflammation

As described above, inflammation plays vital roles in every OTM phase and OIIRR. Although plenty of studies have described the close connection between ferroptosis and inflammation, their inter-regulation effects under different situations remain unclear. Here, the latest investigations on both ferroptosis and inflammation are introduced.

#### 4.1.1. The Connection Between Ferroptosis and Inflammation

The GO and Kyoto Encyclopedia of Genes and Genomes (KEGG) pathway analyses have revealed that a total of 82 corresponding ferroptosis-related differentially expressed genes (FRDEGs) were closely associated with the immune inflammation in acute pancreatitis (AP) [40]. Another in vivo study of mice with AP has shown that Sirtuin4 (SIRT4), a member of the sirtuin family, was involved in inflammation and could inhibit ferroptosis by regulating HIF-1α/heme oxygenase-1 (HO-1) [41].

Sepsis-induced myocardial injury (SIMI) is a widely studied diseases related to ferroptosis-modulated inflammation. Patients with SIMI exhibit elevated levels of inflammatory cytokines, such as TNF-α and IL-6, which could be downregulated by quercetin. Quercetin inhibits ferroptosis by reducing cellular Fe^2+^ and upregulating the level of glutathione peroxidase 4 (GPX4) and ferritin through silencing the SIRT1/p53/Solute Carrier Family 7 Member 11 (SLC7A11) signaling pathway both in vivo and in vitro [42]. Moreover, the ANXA1 small peptide (ANXA1sp) protects against the excessive secretion of proinflammatory cytokines (TNF-α, IL-1β, and IL-6) in SIMI by inhibiting ferroptosis-induced cell death via SIRT3-dependent p53 deacetylation [43]. Furthermore, the m6A methyltransferase (METTL3)-mediated m6A could modify SLC7A11 mRNA, and YTHN6-methyladenosine RNA binding protein 2 (YTHDF2) directly bound to the m6A modification sites of SLC7A11, which promotes the degradation of SLC7A11 mRNA, and results in the upregulation of ferroptosis in SIMI [44].

Respiratory diseases are intricately linked to ferroptosis, as evidenced by various studies. For instance, the upregulation of circEXOC5 exacerbated sepsis-induced acute lung injury (ALI) by stimulating ferroptosis through insulin-like growth factor-2 mRNA-binding proteins 2 (IGF2BP2) recruitment for degrading activating transcription factor 3 (ATF3) mRNA [45]. Moreover, the H1N1 virus can induce the ferroptosis of human nasal epithelial cells (hNECs) by regulating the nuclear factor erythroid 2-related factor 2 (NRF2)/KEAP1/ (glutamate-cysteine ligase catalytic subunit) GCLC signaling pathway, resulting in nasal mucosal epithelial inflammation [46]. In vivo studies have also been conducted to investigate the role of ferroptosis in respiratory diseases. In a rat model of lung ischemia/reperfusion injury, ferroptosis is upregulated and triggers inflammation to further increase lung damage [47]. In C57BL/6 mice, the suppression of Cyclic adenosine monophosphate response element binding protein (CREB) enhances the effect of glucocorticoids on airway inflammation in pediatric asthma by elevating the ferroptosis of eosinophils [48]. In a mouse model of chronic obstructive pulmonary disease (COPD), the deletion of DNA dioxygenase ten-eleven translocation 2 (TET2) triggered ferroptosis and further exaggerated cigarette smoke (CS)-induced inflammation [49].

Ferroptosis also occurs in the regulation of inflammation within macrophages. For example, GTP cyclohydrolase 1(GCH1) could reduce lipopolysaccharide (LPS)-stimulated macrophage polarization and inflammation by protecting the cells from ferroptosis [50]. In addition, the TNF-α autocrine-paracrine loop in ferroptotic macrophages can suppress osteogenesis in mouse bone marrow stromal cells (BMSCs) by regulating the NRF2/FSP1/ROS signaling pathway, which results in bone loss in apical periodontitis (AP) [51]. Zinc oxide nanoparticles (ZnONPs) play an antibacterial role against Mycobacterium tuberculosis (M. tb)-induced inflammation both in vitro and in an in vivo mouse model by enhancing ferroptosis in macrophages to alleviate ALI [52]. Furthermore, hepatocellular ferroptosis-derived oxidative DNA damage actives the stimulator of interferon genes (STING) signaling pathway in macrophages to facilitate the development of liver injury, fibrosis, and tumorigenesis [53].

Interferon regulatory factor 9 (IRF9) has a crucial role in inflammation throughout the progression of bone disease. The knockdown of IRF9 has been shown to inhibit ferroptosis in vitro by activating signal transducer and activator of transcription 3 (STAT3), which subsequently promotes osteoclastogenesis [54]. In an in vivo model of osteoarthritis (OA), the upregulation of ferroptosis, regulated by Forkhead box O3 (FOXO3) via the NF-κB/MAPK signaling pathway, accelerates the progression of OA [55]. Moreover, Cardamonin (CAD) has been found to alleviate OA cartilage degradation by downregulating ferroptosis through the P53 signaling pathway both in vitro and in vivo [56].

In angiotensin II (AngII)- and 2K1C-treated mice, the protein STING interacts directly with the D53 and K412 amino acids of long-chain acyl-CoA synthetase 4 (ACSL4) This interaction can induce renal inflammatory response and fibrosis as a result of ACSL4-dependent ferroptosis. Thus, targeting the STING/ACSL4 axis may be a potential approach to treating hypertension-associated chronic kidney disease (CKD) [57]. Furthermore, the Hippel-Lindau (VHL)/BICD2/STAT1 axis has been demonstrated to inhibit renal inflammation in crystal kidney injury and to increase cell sensitivity to ferroptosis [58].

Some other inflammatory diseases such as diabetes mellitus (DM), cystitis, and hippocampal neuroinflammation have also been linked to ferroptosis. For instance, Arachidonic acid 15-lipoxygenase (ALOX15) contributes to both pathophysiological processes of inflammation and ferroptosis, making it a potential target of DM [59]. Moreover, ferroptosis is associated with cyclophosphamide (CYP)-induced cystitis, and targeting ferroptosis presents a promising method to treat CYP-induced cystitis [60]. Furthermore, maternal sleep deprivation (MSD)-induced neuroinflammation damage in the offspring can be alleviated following treatment with ferroptosis inhibitors [61].

Certain drugs have the ability to induce ferroptosis, which in turn can modulate inflammation. In benzene-exposed mice, ferroptosis is upregulated in benzene-induced hematopoietic toxicity by mediating Th2-type systemic inflammation, and ROS accumulation is a factor in this process [62]. Monobutyl phthalate (MBP) induces ferroptosis that is harmful to the male reproductive system in TM3 cells by modulating the TNF/IL-6/STAT3 signaling pathway [63].

In summary, the upregulation of ferroptosis is associated with increased inflammatory levels in various diseases, and inhibiting ferroptosis of the cells/animals may lead to the amelioration of the diseases. Thus, ferroptosis and inflammation may have close interrelations during the development of diseases. The studies described above have been summarized in Table 1, providing an overview of the current understanding of the relationship between ferroptosis and inflammation in different disease contexts.

#### 4.1.2. Infection-Induced Inflammation and Ferroptosis

Close attention should be paid to those periodontitis patients who need to undergo orthodontic treatment, as the combination of periodontal-related inflammation and orthodontic-induced aseptic inflammation may result in a higher incidence rate of accelerated attachment loss and exacerbate disease progression.

Increasing evidence has demonstrated that ferroptosis is involved in the inflammatory processes of periodontitis both in vitro and in vivo [64].This discovery holds significant promise for improving the diagnosis of periodontitis [65]. A differential expression analysis was conducted and activated B cells were found to exhibit the strongest positive correlation with certain ferroptosis-related genes (FRGs), while macrophages had a strong negative correlation. Importantly, XBP1 and ALOX5 might be crucial genes in the immune-related network in periodontitis [66]. In addition, a ferroptosis-related ceRNA network has been established in periodontitis research, with seven FRGs like IL1B, hsa-miR-185, hsa-miR-204, hsa-miR-211, and hsa-miR-4306, and 28 lncRNAs found to play an important role in the progression of periodontitis [67]. Moreover, LINC00616 has been proven to act as a ceRNA to promote the ferroptosis of PDLSCs via the miR-370/TFRC axis, and the inhibition of LINC00616 promoted cell viability and suppressed the ferroptosis of PDLSCs [68]. Furthermore, butyrate-induced NCOA4-mediated ferritinophagy and ferroptosis may contribute to the loss of periodontal ligament fibroblasts (PDLFs) during periodontitis development [69].

Tetracycline antibiotics, classical drugs for the treatment of adolescent periodontitis, exhibit strong iron-chelating activity [70], suggesting that reducing excessive iron levels may be beneficial in the treatment of periodontitis. Several studies have explored potential drugs to prevent the initiation of ferroptosis in periodontitis, which can broadly be categorized into two groups: iron chelators and antioxidants. For example, deferoxamine, an iron chelator that binds to free iron ions, could inhibit the growth of periodontal pathogens and reduce bone resorption in periodontitis induced by iron overload [71]. Moreover, the usage of the antioxidant enzyme Peroxiredoxin 6 has exhibited inhibitory effects on lipopolysaccharide-induced inflammation and ferroptosis in periodontitis [72].

Although some studies have elucidated the relationship between ferroptosis and periodontal inflammation to some extent, the specific mechanism and key molecules in the relationship between ferroptosis and periodontitis are not fully understood. Many questions still need to be addressed. For example, the accumulation of ROS and iron can also induce cell apoptosis and autophagy. Therefore, it is crucial to explore whether there are any interconnections between ferroptosis and these other forms of cell death. Additionally, determining which form of cell death is dominant and identifying the essential trigger molecule is essential. Further investigations are urgently required to answer these questions.

To sum up, ferroptosis is involved in infection-induced inflammation, like periodontitis. While there is no direct evidence linking orthodontic treatment to ferroptosis, a high failure rate of orthodontic treatment has been observed among periodontitis patients. Therefore, orthodontists must exercise caution and adopt specific methods to treat patients with periodontitis to prevent the worsening of periodontal status due to excessive inflammation, and the application of drugs that inhibit ferroptosis may be helpful. Table 2 summarizes studies that have investigated the relationship between ferroptosis and periodontitis.

### 4.2. The Regulatory Role of Ferroptosis in Bone Homeostasis

**Figure 2:**
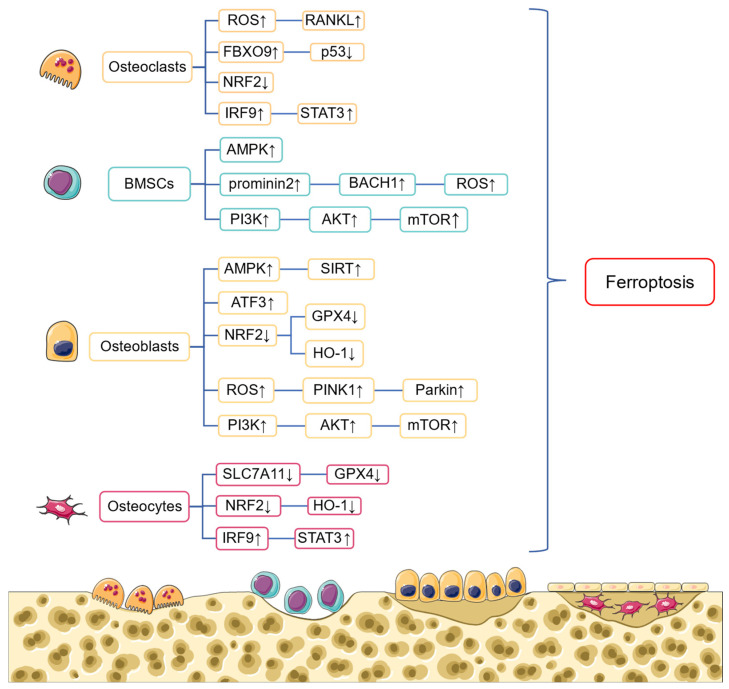
The signaling pathways that trigger ferroptosis in different kinds of bone cells. Bone cells including osteoblasts, osteoclasts, bone marrow mesenchymal stem cells (BMSCs), and osteocytes can undergo ferroptosis through modulating different signaling pathways. Up arrows represent upregulation (↑), and down arrows represent downregulation (↓).

Maintaining the balance between osteoclastic and osteogenic activities is important for keeping the homeostasis and integrity of bone tissue. Osteoclasts are responsible for bone resorption, while osteoblasts are involved in bone reconstruction, including bone formation, mineralization, and the construction of osteocytes. However, it remains unclear whether iron overload and lipid peroxidation affect bone cell activity and disrupt the delicate balance between bone resorption and construction. Here, we summarize studies over the past five years and elucidate the possible relationship between ferroptosis and bone cells, including osteoblasts, osteoclasts, bone marrow mesenchymal stem cells, and osteocytes. Hopefully, the conclusion of this review provides a solid foundation for maintaining bone homeostasis by targeting ferroptosis (Figure 2).

#### 4.2.1. Ferroptosis and Osteoblasts (OBs)

The potential mechanisms and agents that regulate ferroptosis in OBs have been investigated. High-glucose (HG)-induced osteoporosis is one of the most prevalent topics. Studies suggest that advanced glycation end products can induce OB ferroptosis [73], and vitamin K2 inhibits glucose-mediated ferroptosis by activating the AMPK/SIRT1 signaling pathway, resulting in the amelioration of type 2 diabetic osteoporosis (T2DOP). Moreover, vitamin D receptor (VDR) activation by VDR activator (1,25(OH)_2_D_3_) exerts an anti-ferroptosis effect in OBs by the activation of the NRF2/GPX4 signaling pathway [74]. In addition, activating ATF3 contributes to the ferroptosis of OBs and T2DOP pathogenesis [75].

OBs under high-fat conditions experience ferroptosis as well. A recent study suggests that a high-fat environment can induce ferroptosis in OBs by activating the METTL3/apoptosis signal-regulating kinase 1 (ASK1)/p38 signaling pathway, and inhibiting ferroptosis effectively improves OB differentiation and mineralization capacity [76]. Similarly, results have shown that a high-fat diet causes the development of femoral bone loss in mice, which can be inhibited by the ferroptosis inhibitor Fer-1 [77].

Mitochondrial ferritin (FtMt) can regulate cell ferroptosis by storing iron ions and intercepting toxic ferrous ions in mitochondria. A study in a T2DOP rat model has demonstrated that inhibiting FtMt could induce the mitophagy of the bone tissue by regulating the ROS/PINK1/Parkin signaling pathway, while upregulating FtMt could mitigate the risk of ferroptosis in OBs [78].

Exosomes also participate in regulating ferroptosis in OBs. The exosomes secreted by vascular endothelial cells have been shown to regulate osteoblastic ferroptosis, further suppressing ferritinophagy and limiting ferroptosis in OBs [79]. Similarly, in a murine model of osteoporosis, exosomes derived from endothelial progenitor cells have been found to downregulate steroid-induced osteoporosis by inhibiting the ferroptotic signaling pathway [80].

Dexamethasone is closely associated with bone damage. Studies have reported that dexamethasone can activate ferroptosis in OBs by increasing the intracellular Fe^2+^ and ROS levels [81]. On the contrary, melatonin has been shown to alleviate ferroptosis in OBs and enhance their osteogenic properties by stimulating the Nrf-2/HO-1 signaling pathway [82]. Moreover, melatonin also inhibits OB ferroptosis by activating the PI3K/AKT/rapamycin kinase (mTOR) signaling pathway, thereby inhibiting the occurrence of steroid-induced osteoporosis (SIOP) [83]. Furthermore, melatonin reverses the iron-inhibited canonical WNT signaling pathway to restore OB differentiation by downregulating ROS and lipid peroxidation products. This action prevents ferroptosis without causing iron overload in excessive iron-induced osteoporosis [84].

#### 4.2.2. Ferroptosis and Osteoclasts (OCs)

Osteoclasts (OCs) are large, multinucleated cells that are unique in their ability to resorb bone. They are generated from the fusion of mononuclear progenitors of the monocyte/macrophage family. The differentiation of OCs is associated with ferroptosis, and the differentially expressed genes (DEGs) related to their functions are enriched in fatty acids, ROS metabolism, and oxidoreductase activity involving metal ions [85].

RANKL is the key target in regulating bone resorption through ferroptosis. High iron levels can induce osteocyte apoptosis and RANKL production, leading to an increase in the RANKL/OPG ratio in osteocytes, which ultimately enhances the differentiation and bone-resorbing function of OCs [86]. The mononuclear macrophage lineage can differentiate into OCs with the stimulation of RANKL, and ROS plays an essential role in this process. Moreover, ROS produced by iron overload increase the expression level of RANKL and bone resorption [87].

Zoledronic acid, which is currently the first-line anti-osteoporotic drug, inhibits OC differentiation and induces OC apoptosis. Additionally, it has been shown to be involved in OC ferroptosis [88]. It can induce OC ferroptosis by targeting F-box protein 9 (FBXO9)-mediated p53 ubiquitination and degradation [89].

Some transcription factors, such as NRF2 and STAT3, are associated with OC ferroptosis. It has been reported that iron ions can promote OC differentiation and bone resorption by inducing ROS [90], and reducing intracellular iron through NRF2 activation can counteract this effect [91]. In an ovariectomized mouse model of osteoporosis, knocking out IRF9 has been shown to promote OC differentiation by downregulating ferroptosis, and this process involves the activation of STAT3 [54].

#### 4.2.3. Ferroptosis and Bone Mesenchymal Stem Cells (BMSCs)

The low viability and inferior osteogenic differentiation of BMSCs are vital etiologies of osteoporosis. Through a variety of bioinformatics methods, a study has found and identified five differentially expressed genes in vivo that are involved in ferroptosis-related primary osteoporosis in BMSCs, including heat shock protein family A (Hsp70) member 5 (HSPA5), SIRT1, hypoxia inducible factor 1 subunit alpha (HIF1A), mechanistic target of mTOR, and beclin 1 (BECN1). The findings of this study provide some insights into the differentiation mechanisms of BMSCs and may help to elucidate the underlying causes of osteoporosis [92].

There are some signaling pathways involved in the ferroptosis of BMSCs, including AMPK, BACH1, PI3K, and mTOR. A study has indicated that the ferroptosis signaling pathway was significantly activated in BMSCs treated with cigarette smoke extract, as ROS accumulation promotes ferritinophagy via the AMPK signaling pathway, and finally results in ferroptosis [93]. Moreover, the ferroptosis inhibitor ferrostatin-1 can improve BMSC survival by inhibiting ferroptosis, and AMPK might be involved in this process [94]. BMSCs are also great cellular candidates for wound healing. δ-Tocotrienol (δ-TT)-stimulated BMSCs can promote wounding healing in mice by inhibiting BTB and the CNC homology 1 (BACH1) signaling pathway [95]. Another study has revealed the modulatory role of BACH1 in tert-butyl hydroperoxide-induced BMSC ferroptosis, suggesting that targeting the prominin2/BACH1/ROS axis may be a novel way to improve the survival of transplanted BMSCs in clinical practice [96]. The ferroptosis in BMSCs was stimulated after H_2_O_2_ treatment, in parallel with the upregulation of phosphorylated PI3K, AKT, and mTOR [97]. In contrast, tocopherol protects BMSCs from ferroptosis by downregulating the PI3K/AKT/mTOR signaling pathway [98]. In addition, BMSCs can alleviate ferroptosis in rats suffering from testicular damage induced by Cr (VI) via upregulating the phosphorylation of AKT and mTOR. Therefore, BMSC transplantation might become an alternative choice for testicular damage induced by Cr (VI) [99].

A study has confirmed that BMSC-derived exosomes containing miR-223-3p could downregulate ferroptosis by promoting SLC7A11 expression, thereby protecting podocytes from ferroptosis [100]. Likewise, BMSC-derived exosomes carrying miR-367-3p cause a significant decline in microglia ferroptosis by repressing EZH2 and increasing SLC7A11 expression, alleviating the severity of experimental autoimmune encephalomyelitis (EAE) [101]. Moreover, exosomal CircRNA-itchy E3 ubiquitin protein ligase (circ-ITCH) derived from BMSCs inhibits ferroptosis by upregulating NRF2 via recruiting TATA-Box-binding protein associated factor 15 (TAF15) [102]. Furthermore, BMSCs-derived exosomes contain high levels of the lncRNA Mir9-3 host gene (Mir9-3hg), and BMSC-derived exosomes attenuate (I/R)-induced cardiac injury by inhibiting cardiomyocyte ferroptosis through modulating the pumilio RNA binding family member 2 (Pum2)/peroxiredoxin 6 (PRDX6) axis [103].

#### 4.2.4. Ferroptosis and Osteocytes (OCTs)

Osteocytes (OCTs) are the most abundant cell type within mineralized bone tissue. They communicate with other bone cells, including OBs and OCs, through various hormones and the lacunar–canalicular system. Any stimuli that cause OB death or their poor condition can ultimately lead to bone loss and the destruction of bone homeostasis. Therefore, improving the OCTs’ survival rate is essential for maintaining the homeostasis of bone.

Compelling studies have revealed the role of ferroptosis in an OCT precursor cell type, MC3T3-E1. An RNA sequencing study has shown that HO-1 is overexpressed in ferroptotic osteocytes, suggesting a possible function of HO-1 in OCT ferroptosis. In murine models of diabetic osteoporosis (DOP), the promoter activity of HO-1 was controlled by upstream NRF2 and c-JUN transcription factors [104]. In addition, the SLC7A11/GPX4 axis is also involved in diabetic periodontitis-induced alveolar-osteocyte ferroptosis [105]. High glucose and high fat triggered ferroptosis and suppressed the differentiation and mineralization abilities of MC3T3-E1 cells via the activation of the (methyltransferase-like 3)/ASK1-p38 signaling pathway [76].

### 4.3. The Connection Between Ferroptosis and Autophagy

Autophagy has been shown to participate in OTM by acting on different kinds of bone cells [106,107,108,109]. Previously, ferroptosis was thought of as a type of PCD that had no relation to autophagy. However, with increased research, researchers have found that autophagy can be both active or inhibit ferroptosis under different circumstances, and the bridge between these can be ROS, iron accumulation, and lipid peroxidation [110]. Therefore, we hypothesize that the autophagy-related signaling pathway or key molecules may cause OIIRR and as a result, represent targets for modulating ferroptosis in bone cells.

In summary, many studies have demonstrated that four bone-formation-related cells (i.e., osteoblasts, osteoclasts, BMSCs, and osteocytes) experience ferroptosis under various conditions, including those involving autophagy. This process can significantly influence the development and remodeling of bone tissues. Therefore, targeting ferroptosis could be an alternative approach for maintaining bone homeostasis.

## 5. Possible Application of Ferroptosis on Orthodontics

### 5.1. Potential Applications in Preventing Root Resorption and Enhancing Bone Healing

As another hard tissue in the body, the tooth, especially the tooth root, exhibits many similar characteristics to bone. The tooth root contains the dentin around the pulp and the cementum covering the surface of dentin. The odontoblast is the main cell that forms dentin and the cementum is mainly formed by cementoblasts. Ferroptosis occurs frequently in bone-formation-related cells; however, it remains unclear whether odontoblasts and cementoblasts undergo ferroptosis as well, although both the tooth root and the bone can suffer resorption during orthodontic treatment.

Even so, the potential applications of ferroptosis in preventing orthodontic root resorption are worth exploring. For example, an inappropriate magnitude of orthodontic force can cause adverse effects such as excessive hyalinization, uncontrolled tipping, root resorption, and even tooth exfoliation. Researchers are trying to ascertain the optimal orthodontic force (OOF), but a consensus has not yet been reached. Some key molecule expression levels may change in the process of ferroptosis, and this variation could act as an index to find the OOF. Moreover, OIIRR is a common complication during orthodontic treatment, and targeting ferroptosis could be an optimal method to prevent this complication, or provide biomarkers of OIIRR and assist orthodontists in detecting and reducing OIIRR levels. From another perspective, inflammatory mediators can accelerate bone healing by regulating osteoclasts and osteoblasts, causing regional accelerating phenomenon (RAP) after fractures and surgeries such as osteotomies or bone grafting. Tooth movement after surgical treatments (e.g., corticision, corticotomy, and piezocision) might be accelerated by RAP as well. For example, periodontally accelerated osteogenic orthodontics (PAOO) could accelerate bone metabolism and OTM with the performance of selective alveolar corticotomy. As we discussed earlier, ferroptosis and inflammation may have close interactions during bone cell development; hence, ferroptosis is possibly involved in this RAP process. Thus, controlling ferroptosis may be an effective approach to facilitate the successful progression of PAOO.

### 5.2. Possible Applications of Ferroptosis Inhibitors in Orthodontics

In addition, numerous recent studies have linked ferroptosis to the disorders of bone mentioned above, suggesting that modulating ferroptosis may offer a potential treatment for OIIRR. At present, common ferroptosis inhibitors mainly target two key characteristics of ferroptosis: ferrous ion overload and lipid peroxidation. These inhibitors function by reducing free iron, eliminating free radicals, and inhibiting lipid peroxidation [111]. For instance, ciclopirox olamine (CPX-O) and deferoxamine (DFO) are two widely used iron chelation agents. CPX-O has been reported as a promising candidate for Alzheimer disease (AD treatment) by inhibiting intracellular iron accumulation [112], although further clinical trials are needed to confirm these preclinical findings. Its effects on bone diseases have not been studied yet. On the other hand, DFO has been proven effective for postmenopausal osteoporosis (PMOP) through the activation of the KEAP1/NRF2/HMOX1 signaling pathway [113]. In addition, Ferrostatin-1 (Fer-1) is another widely used ferroptosis inhibitor that functions by inhibiting lipid peroxidation. It can regulate ferroptosis and autophagy in mouse osteoblasts (MC-3T3) under mechanical force and promotes bone proliferation [114]. Interestingly, some antioxidants that can trap endogenous free radicals can also serve as ferroptosis inhibitors. In addition to melatonin, which we mentioned earlier, vitamin E (VE) lowers Fe^3+^ in LOX-15 to prevent the synthesis of lipid peroxide [115], and vitamin K (VK) reductase Ferroptosis Inhibitory Protein 1 (FSP1) depletes NAD(P)H to prevent lipid peroxidation and inhibits ferroptosis by converting VK to VKH2 [116].

Until now, no ferroptosis inhibitors have been reported for clinical use in treating OIIRR. The reasons are as follows: Firstly, there is a lack of basic research exploring the efficacy of ferroptosis inhibitors in the treatment of bone diseases. Secondly, a number of agents have been demonstrated to suppress ferroptosis in cell and animal studies; however, due to their poor solubility, low stability, low targeting efficiency and activity, toxicity and poor pharmacokinetics, very few of these medications have made it into human trials. For instance, DFO exhibits low solubility and targeting efficiency [117], and Fer-1 has low solubility and stability [118]. Thirdly, some ferroptosis inhibitors such as melatonin and VE have already been used in humans, but they still present challenges in clinical studies, because of the severe side effects or the inconsistent evidence for their curative effect [119].

Despite these challenges, the potential of ferroptosis modulation as a therapeutic strategy for OIIRR remains promising. Future research should focus on identifying more effective and safer ferroptosis inhibitors, as well as optimizing their delivery and targeting to bone tissues. Additionally, it is crucial to conduct comprehensive preclinical and clinical studies to validate the efficacy and safety of these inhibitors in treating OIIRR and other bone disorders.

## 6. Perspectives and Conclusions

An increasing number of studies have shown the interactions between ferroptosis and bone homeostasis/regeneration. The accumulation of iron and oxidative stresses is associated with abnormal bone metabolism, suggesting that targeting ferroptosis might be a promising approach for combating OIIRR. In this review, we elucidate the concept of ferroptosis and the mechanisms underlying OTM, and place a particular emphasis on the relationship between inflammation and ferroptosis. We also highlight the regulatory function of ferroptosis in maintaining bone homeostasis, shedding light on how this form of cell death can influence bone resorption and formation. Notably, we proposed potential applications of ferroptosis in modulating OIIRR.

However, many problems still need to be solved in order to achieve this. For instance, since various types of cell death are closely entangled in tooth resorption, it is difficult to clarify the mechanism by which the cells choose to die as ferroptosis and how the cells survive it. The determining factors for the cells to induce or survive ferroptosis in OIIRR remain unknown as well. Moreover, although ferroptosis is involved in bone homeostasis and inflammatory diseases like periodontitis, there is currently no standardized testing technology that meets the clinical diagnostic needs. Therefore, more suitable biomarkers need to be discovered for the prevention and prognostication of OIIRR. Furthermore, ferroptosis is distinguished from other types of cell death including apoptosis and autophagy, yet some ferroptosis inducers like erastin and key regulators of ferroptosis such as GPX4, SLC7A11, P53, NRF2, HO-1, PI3K/AKT/mTOR, and STAT3 are implicated in apoptosis or autophagy as well. Understanding the interactions among these signaling pathways may provide insights into the advancement of studies on, and clinical interventions for, OIIRR. In addition, ferroptosis inhibitors have not been applied in clinical settings for the treatment of bone diseases, hence further studies to find targeted ferroptosis inhibitors and clinical studies are urgently needed.

Therefore, further research is warranted to elucidate the specific mechanisms controlling ferroptosis in the initiation and progression of OIIRR. Such research will pave the way for the development of innovative and promising strategies for the clinical prevention, diagnosis, and treatment of OIIRR. In the near future, the relationship between ferrous and ROS metabolism and bone homeostasis might become a promising research area. The hope is to find a multi-link, multi-target, comprehensive approach to preventing OIIRR during OTM.

## Figures and Tables

**Table 1:** The signaling pathways that trigger ferroptosis in inflammatory diseases.

Diseases	Signaling Pathways	Ref.
Acute pancreatitis (AP)	HIF-1α/HO-1	[41]
Sepsis-induced myocardial injury (SIMI)	SIRT1/p53/SLC7A11; SLC7A11 expression level; N6-methyladenosine writer METTL3	[42,44]
Sepsis-induced acute lung injury (ALI)	IGF2BP2 recruitment and ATF3 mRNA expression level	[45]
Nasal mucosal epithelial inflammation	NRF2/KEAP1/GCLC	[46]
Chronic obstructive pulmonary disease (COPD)	Expression level of DNA dioxygenase TET2	[49]
Apical periodontitis (AP)	NRF2/FSP1/ROS	[51]
Osteoarthritis (OA)	FOXO3/NF-κB/MAPK	[55]
Chronic kidney disease (CKD)	STING/ACSL4	[57]
Crystal kidney injury	VHL/BICD2/STAT1	[58]
Maternal sleep deprivation (MSD)-induced neuroinflammation damage in the offspring	Nrf2 and HO-1 expression level	[61]

**Table 2:** The different types of studies that describe the relationship between ferroptosis and periodontitis.

Study Object	Study Types	Mechanisms	Ref.
Healthy samples and patients with periodontitis	Bioinformatics analysis	B cells are positively correlated with FRGs, T cells are negatively correlated with FRGs	[66]
mRNA expression profiles between periodontitis and healthy gingival tissues	Bioinformatics analysis	Seven ferroptosis-related FRGs, IL1B, hsa-miR-185, hsa-miR-204, has-miR-211, and hsa-miR-4306, and 28 lncRNAs are related to periodontitis	[67]
Periodontal ligament stem cells (PDLSCs)	In vitro	LINC00616 promotes ferroptosis via the miR-370/TFRC axis	[68]
Primary human periodontal ligament fibroblasts (PDLFs)	In vitro	Butyrate-induced NCOA4-mediated ferritinophagy and ferroptosis contribute to the loss of PDLFs	[69]
Human periodontal ligament cells (hPDLCs)	In vitro	Deferoxamine inhibits the growth of periodontal pathogens and reduces bone resorption	[71]
Both inflamed and non-inflamed human gingival tissues	Human research	Peroxiredoxin 6 inhibits lipopolysaccharide-induced inflammation and ferroptosis	[72]
